# Integrative and syntactic complexity’s role in decision-making under uncertainty

**DOI:** 10.3389/fpsyg.2024.1450703

**Published:** 2025-01-03

**Authors:** Alejandra Mitzi Castellón-Flores, Edmundo Molina-Perez, Isaac Molina, Pedro Manuel Cortes, Fernanda Sobrino, Luis Serra-Barragan

**Affiliations:** School of Government and Public Transformation, Tecnologico de Monterrey, Monterrey, Mexico

**Keywords:** decision making, integrative complexity, syntactic complexity, uncertainty, rational decisions

## Abstract

This study explores the impact of integrative complexity (IC) and syntactic complexity (SC) on decision-making under uncertainty. The research addresses how cognitive structures contribute to decision quality in ambiguous situations. A modified Ellsberg experiment was conducted using an online platform. Participants were exposed to varying levels of ambiguity, and decision support tools were introduced to assess the influence of IC and SC on decision-making. The manipulation of available information allowed for a controlled analysis of cognitive processing. The findings reveal that IC and SC significantly enhance decision quality. IC facilitates the integration of diverse information, while SC supports the comprehension and management of ambiguity. Both cognitive structures play essential roles in navigating uncertainty. These results underscore the importance of IC and SC in effective decision-making. The findings suggest that fostering these cognitive abilities may improve decision-making skills in uncertain contexts, offering practical implications for training and development in high-stakes environments.

## 1 Introduction

Decision-making under uncertainty is a fundamental area of research at the intersection of cognitive psychology, behavioral economics, and decision theory. The ability to make informed and rational decisions when faced with ambiguity or uncertain risk is crucial in numerous contexts, from business management to public policy and individuals’ everyday lives.

Despite advances in understanding the mechanisms guiding decision-making under uncertainty, there remains a gap in how cognitive structure influences these processes. Two key concepts that have gained prominence in this field are integrative complexity (IC) and syntactic complexity (SC). IC refers to an individual’s ability to differentiate and then integrate multiple dimensions of information into a coherent judgment. In contrast, SC examines how thoughts and expressions are structured in an organized and elaborate manner, potentially influencing how complex information is processed and communicated.

While much research has focused on IC’s role in decision-making, there is a notable lack of studies exploring SC’s contribution, particularly in contexts involving ambiguity and risk. SC could play a crucial role in how individuals organize information when faced with uncertain outcomes, yet its relationship with decision-making remains understudied. This study seeks to address this gap by investigating how both IC and SC influence decision-making under uncertainty.

We hypothesize that higher levels of both IC and SC will be associated with improved decision-making outcomes in uncertain scenarios. Specifically, individuals who exhibit greater integrative and syntactic complexity are likely to process ambiguous information more effectively, leading to more informed and adaptable decisions.

## 2 Background and related work

Before delving into the specifics of our experiment, it is crucial to establish a foundation of key concepts.

### 2.1 Ellsberg Paradox

The Ellsberg (1961) is a landmark in the study of decision-making under uncertainty, revealing individuals’ tendency to avoid ambiguity by preferring options with known risks over those with unknown risks, even when the probabilities involved are objectively equivalent. This paradox challenges traditional models such as Expected Utility Theory (von Neumann and Morgenstern, 1944), which assumes that rational decision-makers base their choices purely on the expected outcomes of known probabilities.

Ellsberg’s experiment presents a scenario where participants must choose between a gamble with a known probability and another with an unknown one. Despite both options offering mathematically similar chances of success, people overwhelmingly prefer the known probability. This behavior is termed ambiguity aversion (Halevy, 2007), as individuals exhibit a discomfort with the uncertainty surrounding the unknown option, preferring the security of known risks.

The Ellsberg Paradox not only questions the assumptions of economic rationality but also demonstrates that decision-making under uncertainty involves more than probabilistic reasoning—it includes cognitive factors such as perception, emotion, and risk tolerance. Studies have suggested that this aversion to ambiguity can be linked to a psychological mechanism where individuals overestimate the likelihood of negative outcomes when probabilities are unclear (Neace et al., 2011).

Moreover, the paradox has changed the field of behavioral economics, pushing for models that better account for human tendencies, such as ambiguity aversion and cognitive biases, which deviate from the notion of the “perfectly rational” decision-maker (Jia et al., 2020; Kovářík et al., 2016). This shift has been crucial for understanding real-world economic behavior, particularly in high-stakes decisions involving significant uncertainty.

### 2.2 Dynamic and quantum perspectives

Building upon the foundational insights provided by the Ellsberg Paradox, which illustrates individuals’ aversion to ambiguity, more recent theoretical advancements have sought to refine and expand our understanding of decision-making under uncertainty. While Ellsberg demonstrated that individuals tend to favor known risks over ambiguous ones, dynamic and quantum perspectives offer alternative explanations for how these preferences can evolve over time or be modeled more accurately (Aerts et al., 2011).

Dominiak et al. (2009) extended Ellsberg’s framework by incorporating dynamic conditions, revealing that individuals’ preferences are not static. Instead, they adapt as new information becomes available. This adaptation is guided by principles such as dynamic consistency and consequentialism, which suggest that decision-makers revise their choices when they encounter updated probabilities or new data, allowing for more flexible strategies when navigating ambiguity.

Additionally, the quantum decision theory proposed by al-Nowaihi and Dhami (2017) offers another layer of complexity by applying quantum probability to decision-making. Their model posits that uncertainty might be better captured using quantum probabilistic processes, which challenge the traditional assumptions of probability weighting. This model, which introduces projective expected utility, aligns closely with empirical observations of decision-making anomalies like those seen in the Ellsberg Paradox. It provides a parameter-free approach to predicting behavior, suggesting that quantum principles might underlie the cognitive biases individuals exhibit in ambiguous decision contexts.

These dynamic and quantum models both build on and challenge the classical interpretations offered by the Ellsberg Paradox, suggesting that decision-making under uncertainty is a far more fluid and context-dependent process than originally thought. As ambiguity aversion is explored through these new lenses, we gain a richer understanding of how individuals process uncertainty and adjust their choices based on evolving information.

### 2.3 Extending Ellsberg with experimental investigations

Building on the insights of the Ellsberg Paradox, which demonstrated ambiguity aversion, researchers have continued to explore how individuals process ambiguity through various experimental setups. Halevy (2007) contributed to this by introducing a more intricate experimental framework that differentiates between risk and ambiguity, incorporating compound lotteries to investigate how individuals deal with multi-layered uncertainties. His findings confirmed that ambiguity aversion persists in complex decision scenarios, reinforcing the view that decision-making under uncertainty involves not just risk but a deeper cognitive engagement with ambiguity.

More recent work, such as that by Aydogan et al. (2023), further delves into the complexity of ambiguity aversion by conducting experiments to assess how sophistication and complexity influence decision-making under uncertainty. Their results showed that ambiguity aversion remains robust across varying degrees of cognitive complexity, but that the relationship between attitudes towards ambiguity and compound risk can vary. Notably, for individuals with lower cognitive complexity, attitudes toward complexity appear to play a role in shaping their ambiguity aversion. This indicates that how individuals process and respond to ambiguous information is not merely a function of risk but also of their capacity to handle complexity in decision environments.

These findings highlight that ambiguity aversion is not a fixed trait but is influenced by the nature of the uncertainty and the complexity of the decision environment. This has important implications for understanding how ambiguity aversion plays out in real-world scenarios, such as financial markets, where individuals consistently prefer investments with clearer risk profiles, even when the expected returns are similar (Klingebiel and Zhu, 2023). Such research builds on Halevy’s foundational work and offers deeper insights into how decision-making strategies evolve as the complexity of the decision increases.

### 2.4 Exploring integrative complexity

Unlike the dynamic and quantum models that focus on how external information shapes decision preferences, integrative complexity (IC) shifts the focus to the cognitive structures that individuals use to process that information. By understanding IC, we can explore how some decision-makers are able to embrace uncertainty more effectively, synthesizing complex data rather than merely reacting to ambiguity. IC measures the extent to which an individual can differentiate between multiple perspectives and then integrate them into a coherent judgment, making it particularly relevant in decision-making scenarios characterized by ambiguity.

Suedfeld’s research into political decision-making demonstrates that individuals with higher levels of IC are better equipped to process conflicting information in high-pressure situations, such as crises or negotiations. These findings are applicable to decision-making under economic uncertainty as well, where individuals must evaluate ambiguous information and make informed judgments. In contrast to those who exhibit ambiguity aversion, individuals with higher IC can engage with uncertain information, integrating it into their decision-making processes without being overwhelmed by the ambiguity itself.

Further reinforcing this connection between IC and decision-making, Thoemmes and Conway (2007) conducted an analysis of US presidents’ State of the Union addresses to examine changes in IC over the course of their terms. Their findings revealed that IC tends to be highest at the beginning of a president’s first term, gradually decreasing toward the end, especially for presidents who win reelection. This suggests that strategic shifts in rhetoric and cognitive processing occur in response to the demands of leadership and political cycles, highlighting how IC adapts to external pressures over time.

This framework aligns with the insights drawn from Halevy’s (2007) work, as discussed in the previous sections, which revealed that complexity in decision-making environments affects how individuals respond to ambiguity. By incorporating IC into the analysis, we gain a deeper understanding of how individuals navigate the challenges of uncertainty, offering a more comprehensive view of decision-making strategies in uncertain environments.

### 2.5 Distinguishing syntactic complexity

In addition to IC, syntactic complexity (SC) plays a pivotal role in decision-making under uncertainty. SC refers to the degree to which individuals structure their language—whether in written or verbal form—in a complex and organized manner (Jagaiah et al., 2020) High SC is characterized by longer, more intricate sentence constructions that reflect a more elaborate cognitive processing of information, while low SC is typically associated with simpler sentence structures that might indicate less detailed reasoning (Kagan, 1980; Wang et al., 2023). Understanding how individuals structure their thoughts, especially under conditions of uncertainty, can provide valuable insights into their decision-making processes.

Research into SC has expanded significantly in recent years, particularly in the context of ambiguity resolution. Recent studies, such as those conducted by Aydogan et al. (2023) and other researchers, demonstrate that SC can influence how individuals navigate ambiguous situations. These studies show that individuals with higher SC tend to process ambiguity more effectively, breaking down complex scenarios into manageable pieces and arriving at more informed decisions. Similarly, experiments in ambiguity resolution within linguistic contexts also reveal that individuals who exhibit higher SC are better equipped to handle syntactic and contextual ambiguity, leading to more effective decision outcomes (Spotorno et al., 2015).

Moreover, the relationship between SC and working memory has been highlighted as a crucial factor in the ability to resolve ambiguity. Studies show that individuals with higher working memory capacity tend to manage more complex syntactic structures, which in turn aids in the resolution of ambiguities during decision-making (Kim and Christianson, 2013). This suggests that SC not only reflects the complexity of thought but is also closely tied to cognitive resources, such as memory, that are essential for processing uncertainty.

By incorporating SC into the broader framework of decision-making under uncertainty, we can gain a more nuanced understanding of how individuals approach ambiguous situations. High SC allows individuals to organize their thoughts more effectively, enabling them to process multiple layers of uncertainty without becoming overwhelmed (Silva, 2022; Khan et al., 2018). This complements the findings from IC and offers a comprehensive view of cognitive strategies in decision-making.

### 2.6 Integrating cognitive complexity models

Both IC and SC offer valuable insights into the cognitive mechanisms that individuals use when making decisions under uncertainty. While each concept focuses on different aspects of cognitive functioning—IC on the differentiation and integration of perspectives, and SC on the complexity of linguistic expression—there is a growing recognition that these two dimensions are deeply interconnected. Both IC and SC represent different manifestations of cognitive complexity, and together they provide a more comprehensive framework for understanding how individuals navigate ambiguity and uncertainty.

Building upon these insights, Tadmor et al. (2009) explored the cognitive implications of biculturalism, focusing on how acculturation strategies affect IC, a cognitive style that involves acknowledging and integrating multiple perspectives. Their studies revealed that biculturals—according to Tadmor et al. (2009), biculturalism involves the simultaneous maintenance of one’s own cultural heritage while incorporating a new one as part of one’s identity—exhibit higher IC levels across different domains, suggesting that exposure to multiple cultures enhances the ability to process and integrate diverse viewpoints. This enhancement in IC was linked to the biculturals’ ability to navigate between cultural frameworks and merge conflicting cultural perspectives into a cohesive cognitive approach. Recent studies on IC continue to emphasize its critical role in decision-making, highlighting its connection to cognitive flexibility and adaptability, which are essential in high-stakes, uncertain environments (Molina et al., 2023).

On the other hand, recent research on SC has expanded significantly, with studies emphasizing its role in processing linguistic ambiguity and complex decision tasks. SC has been shown to correlate with the ability to break down complex scenarios into manageable components, facilitating more structured decision-making in ambiguous contexts (Jagaiah et al., 2020; Lyu et al., 2022). This integrated approach to studying IC and SC allows for a more detailed understanding of how cognitive structures influence the strategies employed to resolve uncertainty.

Moreover, cognitive complexity models have been shown to play a critical role in adaptive decision-making. Ambiguity aversion, as discussed earlier, often leads to decision avoidance or risk-averse behavior when individuals are confronted with uncertain outcomes. However, individuals with high IC and SC are better equipped to overcome these biases by organizing and synthesizing information in a way that facilitates clarity, even in ambiguous contexts (Lilleholt, 2019). This adaptability is essential in real-world decision-making scenarios, where uncertainty is a constant factor and the ability to manage it effectively can be a critical determinant of success.

By integrating these models, we not only gain a richer understanding of how cognitive processes interact during decision-making under uncertainty but also open new avenues for research into how these processes can be enhanced. Understanding the relationship between IC and SC provides a clearer picture of the cognitive tools that individuals use to approach ambiguity and offers practical applications in fields such as leadership, negotiation, and strategic planning, where decisions often must be made without complete information.

### 2.7 Syntactic and semantic dimensions in integrative complexity

A study by Robertson and Broadhurst (2019) offers a critical perspective on previous approaches to measuring IC, highlighting a significant limitation: the predominant focus on semantic content, with minimal attention to syntactic structures. They argue that capturing how thoughts are structured syntactically is crucial for accurately assessing the cognitive processes underlying IC. IC not only involves recognizing and integrating multiple perspectives but also how these perspectives are organized in the mind and expressed through language.

Earlier automated systems for scoring IC, such as those developed by Ambili and Rasheed (2014) and Conway et al. (2014), focused primarily on semantic markers—specific words or phrases associated with differentiated or integrated thinking. While these models represented significant progress in measuring IC, Robertson and Broadhurst point out that they largely overlooked the syntactic complexity that often underpins sophisticated cognitive processes. For instance, the use of subordinating conjunctions or complex sentence structures can provide valuable insights into higher-order reasoning, revealing the depth of cognitive engagement that goes beyond simple word frequency counts or presence of particular vocabulary.

To address this gap, Robertson and Broadhurst propose an advanced machine learning model that integrates both semantic and syntactic features in assessing IC. This model not only considers the presence of complex vocabulary associated with differentiated thinking but also how these elements are organized within sentences, reflecting syntactic nuances that signal higher-level cognitive processes. For example, the ability to use subordinating conjunctions to link multiple clauses can indicate a more complex, integrative reasoning process that might otherwise be missed by models focusing solely on semantics.

By incorporating syntactic analysis into IC assessment, Robertson and Broadhurst align their methodology more closely with the theoretical foundations of IC, which emphasize not only the differentiation of ideas but also how these ideas are synthesized into a coherent whole. They argue that the syntactic structuring of language—how ideas are framed, linked, and developed in discourse—plays a critical role in cognitive integration.

Further supporting this approach, Meltzer et al. (2010) demonstrate that different brain regions are activated selectively by syntactic complexity and semantic reversibility, underscoring the importance of SC in understanding complex cognitive processing. Their findings show that while semantic reversibility activates broad areas of the brain, syntactic complexity—particularly in reversible sentences—engages the left inferior frontal gyrus (LIFG) and adjacent regions. This evidence highlights that syntactic parsing is not automatic but requires selective engagement depending on the complexity of the sentence and its meaning.

### 2.8 The influence of syntactic complexity on decision-making

In decision-making contexts, where ambiguity and uncertainty are prevalent, the ability to process and integrate SC could significantly influence how decisions are formed and communicated. For example, a decision-maker with high IC, proficient in handling syntactic complexities, might better navigate the ambiguities of complex scenarios through a more structured and nuanced integration of information. This capability could lead to more robust decision-making processes, where various perspectives and data are synthesized more coherently.

This enhanced IC model offers significant potential for application in large-scale text analysis, such as examining communication on social media platforms, where the vast amounts of data make manual scoring impractical. The ability to automatically and accurately gauge complexity of thought in real time opens new avenues for research into cognitive styles across diverse contexts and populations.

The addition of syntactic analysis allows for a more nuanced and theoretically grounded IC assessment. Syntax provides a framework to understand not just the presence of complex ideas but their organization, which is crucial in evaluating how individuals structure their thoughts when dealing with multifaceted issues. For example, a text that uses multiple clauses effectively, with phrases that connect these clauses showing contrast, conditionality, or causality, might indicate higher IC than a text with a similar range of vocabulary but a simpler sentence structure.

In this context, IC and SC emerge as relevant constructs for better understanding the underlying processes in decision-making under ambiguity. IC, which refers to the ability to differentiate and then integrate multiple dimensions of information into a cohesive judgment, and SC, which relates to the ability to structure responses in a syntactically complex manner, are indicators of how cognitive structure can influence the interpretation and response to ambiguous information.

Recent research has begun to explore how these information-processing dimensions affect decision-making, suggesting that higher IC may be associated with a greater ability to navigate uncertainty and make informed decisions (Suedfeld, 2010; Tadmor et al., 2009). However, there is a critical need to further investigate these relationships in the context of explicit economic decisions and how these cognitive capacities modulate individuals’ responses to ambiguity and risk.

Despite their theoretical potential, the relationship between IC, SC, and decision-making in practical ambiguity contexts remains relatively underexplored. Preliminary research suggests that higher IC may enhance decision-making performance, particularly when individuals face complex and uncertain environments that require the integration of multiple perspectives.

However, the specific hypothesis of this study posits that higher levels of both IC and SC together will lead to more effective decision-making under conditions of high ambiguity, where the clarity of information is reduced, and the decision-maker must navigate conflicting or incomplete data. Specifically, SC is expected to influence how well individuals can structure and communicate their reasoning, thereby enhancing the integrative processes captured by IC. The role of SC, especially in conjunction with IC, and its impact on decisions involving ambiguous information presentations, has yet to be thoroughly examined in empirical contexts.

## 3 The present research

The present research seeks to expand on the current understanding of decision-making under uncertainty by focusing on the roles of IC and SC. While existing theories, such as the Ellsberg Paradox, highlight human tendencies toward ambiguity aversion, there remains a significant gap in comprehending how cognitive structures, particularly IC and SC, shape decision-making processes when individuals are confronted with ambiguous information. This study is designed to address this gap by investigating how these cognitive mechanisms contribute to decision-making in environments characterized by uncertainty.

The research aims to explore the extent to which IC enhances an individual’s capacity to integrate complex and often contradictory information. We posit that individuals with higher IC will exhibit a greater ability to synthesize diverse perspectives, leading to more adaptive and informed decision-making, even in situations where the available data is incomplete or ambiguous. IC is hypothesized to play a crucial role in navigating uncertainty by allowing individuals to evaluate multiple dimensions of a problem, thereby facilitating a more coherent and flexible approach to decision-making.

At the same time, this study examines how SC contributes to the structuring of thoughts and communication in uncertain scenarios. Individuals who display higher levels of SC are expected to process and structure ambiguous information more effectively, leading to improved clarity in their decision-making strategies. SC is proposed to help organize complex ideas, thereby enabling participants to manage ambiguity with greater precision and confidence. By structuring their cognitive processes more clearly, these individuals are likely to make more robust decisions when faced with uncertain risks. Additionally, we sought to explore whether there is a relationship between IC and SC scores and other sociodemographic variables, such as potential differences in gender, educational attainment, or age. Our revised hypothesis posits that these sociodemographic factors are unlikely to have a significant impact on IC and SC scores, as the cognitive processes underlying integrative and syntactic complexity are expected to be relatively independent of these external variables. Nevertheless, this study will rigorously investigate these potential relationships to confirm whether sociodemographic differences play any role in influencing IC and SC outcomes.

Moreover, this research investigates the interaction between IC and SC in enhancing decision-making performance. The combination of these two cognitive structures is expected to foster superior decision-making outcomes by promoting both the integration of differentiated perspectives and the clear organization of thought processes. Such synergy is anticipated to enable individuals to navigate ambiguity more effectively and make decisions that are both well-informed and adaptable.

In this context, the study employs a variation of the Ellsberg experiment to manipulate the availability of information and assess how participants handle varying levels of ambiguity. The results are expected to provide valuable insights into the relationship about CI and CS that contribute to effective decision-making and suggest that fostering IC and SC could serve as a strategy for improving decision-making under conditions of risk and ambiguity.

## 4 Outlining the challenge and methods

To work toward filling the gap of knowledge surrounding decision-making under uncertainty, we undertook an experimental investigation. The premise of this work acknowledges that decision-making under uncertainty poses a critical challenge that involves complex cognitive structures and information processing strategies. Notably, the Ellsberg Paradox illustrates individuals’ tendency to avoid ambiguity, preferring options with known risks over those with unknown risks. This behavior defies the principles of traditional economic rationality and indicates the need for decision-making models that accommodate how people perceive and process in ambiguous scenarios.

In particular, deploying decision support tools in controlled environments where uncertainty is manipulated may offer novel insights into how individuals integrate and use computational support to enhance decision-making capabilities. Thus, we must explore how these cognitive dimensions modulate individuals’ responses to ambiguity and risk, and how these capabilities might be enhanced to support more informed and effective decision-making in real-world economic and political contexts.

### 4.1 Methodology

To begin, we programmed the experiment on the Cognition.run platform using the jsPsych package.

### 4.2 Participants

We obtained a total of 2,000 participants who completed 100 percent of the trials. All participants were Mexican, native Spanish speakers from Mexico, and reported no cognitive or neurological conditions.

It is important to note that, in addition to the main sample, 1,823 participants were excluded from the final analysis because they did not complete the task. This decision was made due to the necessity of having complete data from the Ellsberg task and participants’ written responses to conduct meaningful comparisons across task conditions. Ensuring the completeness of both sets of data was essential for the integrity of our analysis. The data collection process, which required obtaining fully completed tasks, spanned a total of 13 months.

### 4.3 Experimental design

Drawing inspiration from the Ellsberg Paradox, we designed an online experiment examining how IC and SC influence decision-making in a modified scenario of this dilemma. Participants were randomly assigned to one of three conditions: the first presented the number of balls per color (green, red, blue) in each container; the second combined the numbers of green and blue balls; and the third maintained this combination but additionally incorporated a decision support tool offering a table with percentage probabilities for each ball color. This design allowed us not only to replicate the classic phenomenon of ambiguity aversion, but also to investigate how the presentation of information and the availability of decision support tools affect information processing and choice under conditions of known and unknown risk and ambiguity.

#### 4.3.1 Stage one: baseline assessment

**Figure 1:**
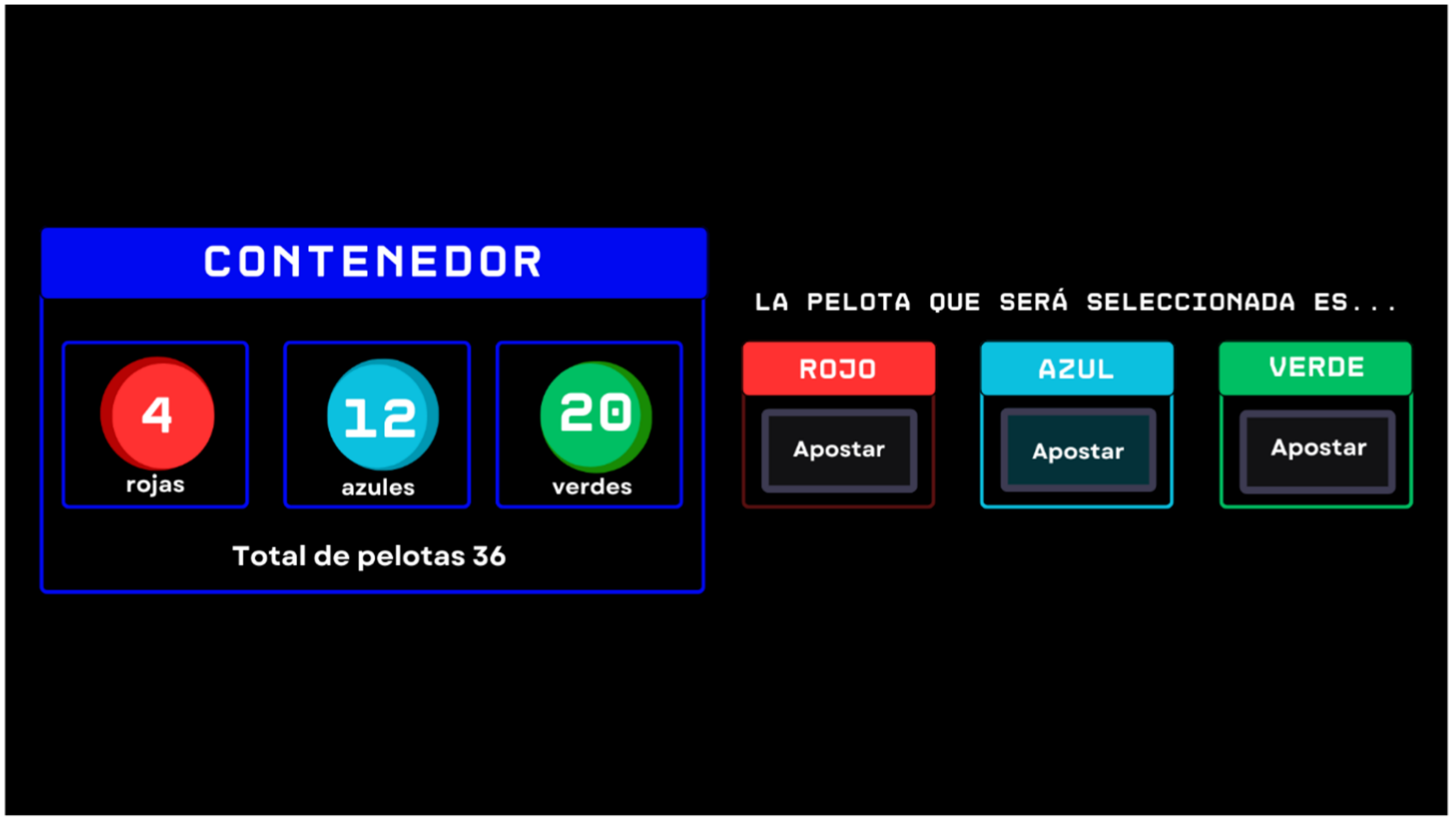
Example of the trial in the stage one: baseline assessment. An example of one of the trials from stage 1 is shown. In the outlined in blue box, the numbers of balls by color in the container are displayed. On the right side, the bet that the participant must place for the color of the ball they believe is most likely to be drawn can be seen.

Participants were presented individually with a digital interface displaying a container filled with red, green, and blue chips. The exact count of chips in each color was provided, offering participants a scenario with complete information. Participants were then tasked with wagering on the color of a chip randomly selected from the container, thereby establishing a baseline for decision-making under certainty. Figure 1 shows an example of what participants saw during the task.

#### 4.3.2 Stage two: introducing uncertainty

**Figure 2:**
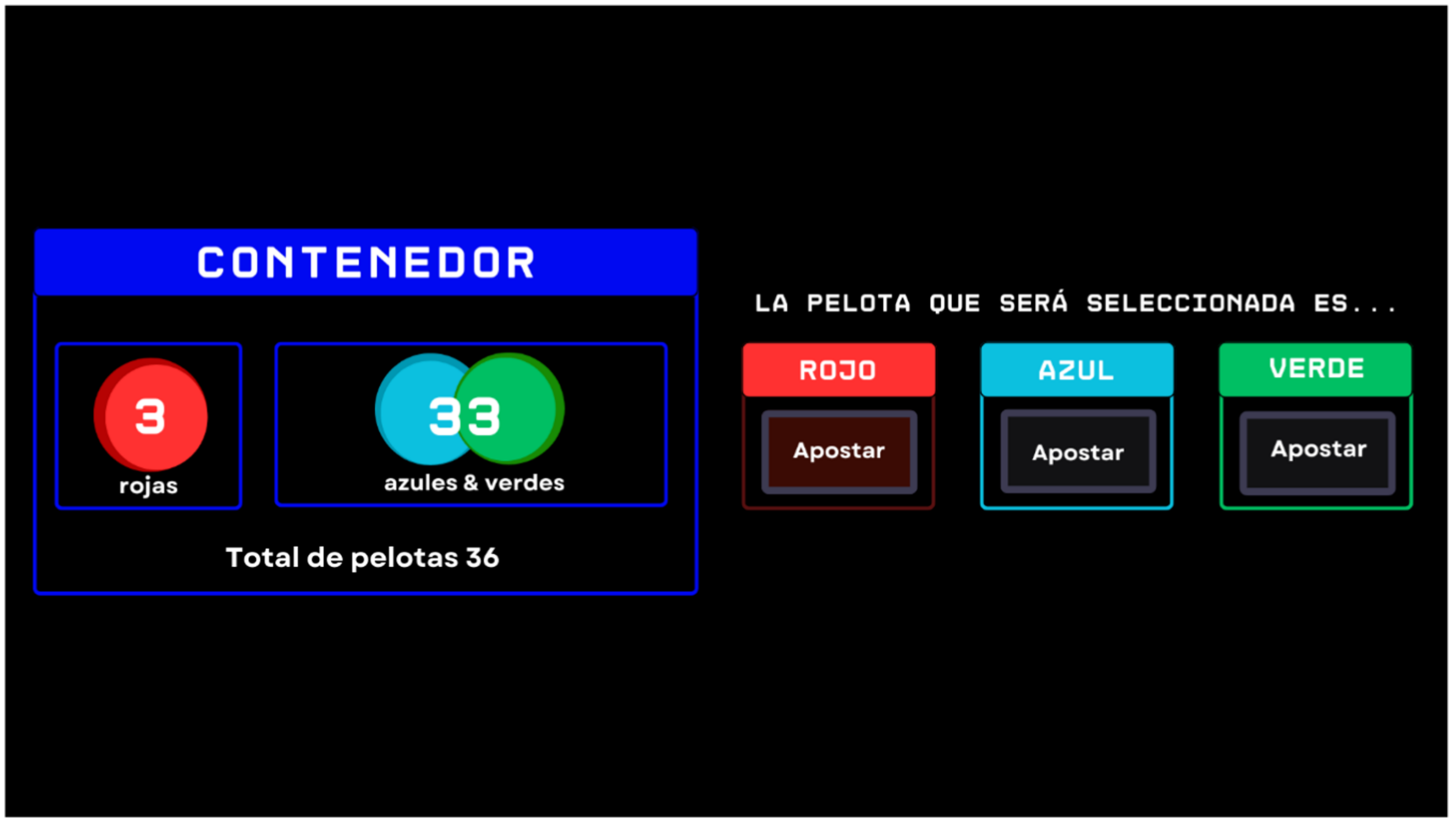
Example of the trial in the stage two: introducing uncertainty. An example of one of the trials from stage 2 is shown. In the box outlined in blue, the number of balls by color in the container is displayed; in this case, the numbers of blue and green balls are combined. To the right, the bet that the participant must place is visible, indicating the color of the ball that they consider most likely to be drawn.

Mirroring the first stage in structure, this phase altered the informational landscape. Participants were informed of the number of red chips but were only given the combined total of green and blue chips, not the specific counts for each. This adjustment introduced a layer of ambiguity, challenging participants to make decisions with incomplete data about the outcome probabilities (see Figure 2).

#### 4.3.3 Stage three: introducing decision support

**Figure 3:**
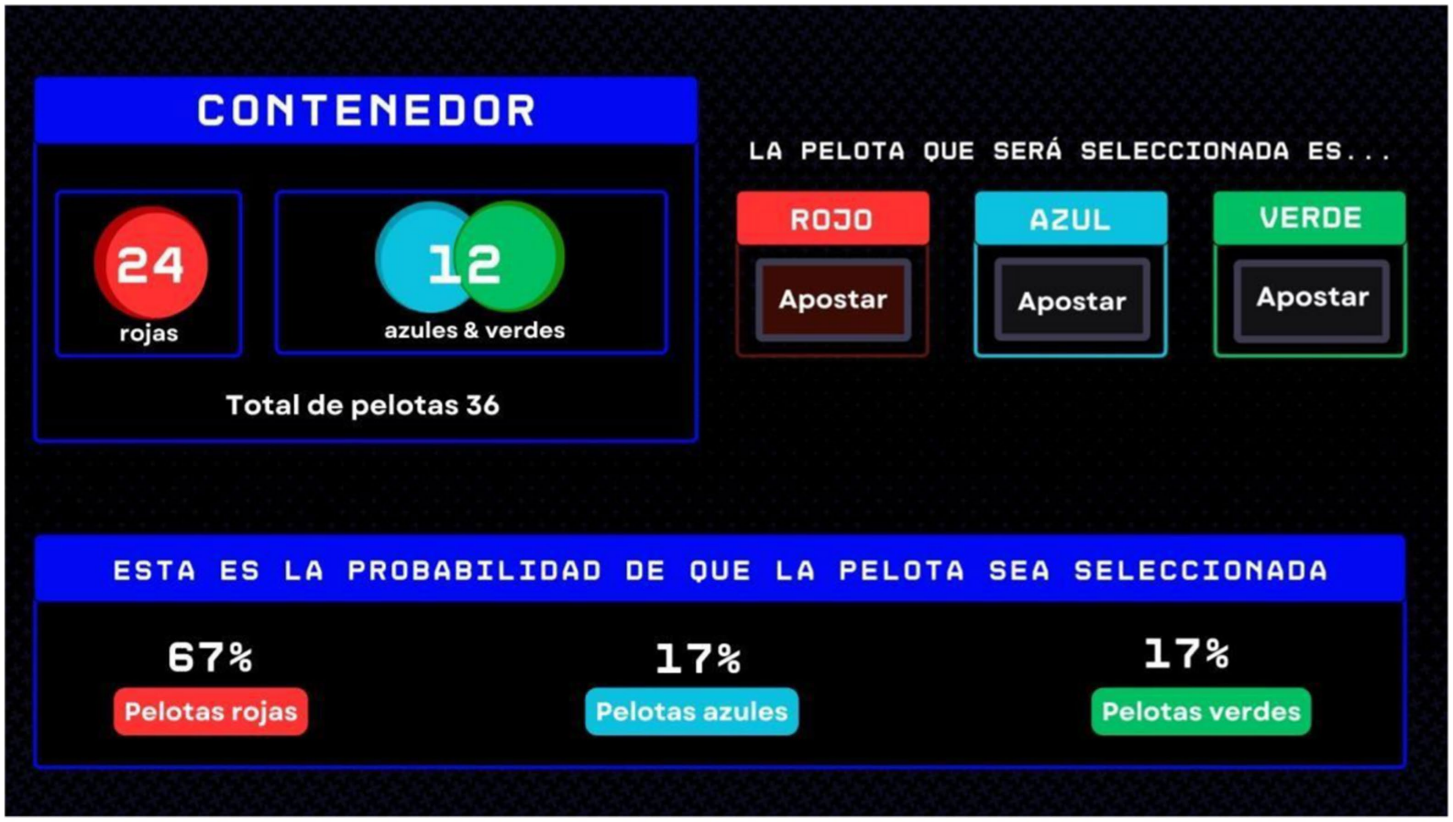
Example of the trial in the stage three: introducing uncertainty. An example of one of the trials from condition 2 is presented. The box outlined in blue displays the number of balls by color in the container; in this case, the numbers of blue and green balls are combined. However, in the central lower part, there is a decision-making tool that indicates the percentage probability of a ball of a specific color being drawn. On the right side, the participant’s bet is shown for the color of the ball they consider most likely to be drawn.

The final stage maintained the uncertainty level established in the second stage, offering no additional clarity on the green- and blue-chip distribution. However, this phase introduced a decision support tool that provided statistical probabilities for each chip color being selected. This tool aimed to examine whether and how computational intelligence influences decision-making strategies and emotional responses when faced with ambiguity (see Figure 3).

### 4.4 Integrative complexity score

IC is a measure that assesses thought processes’ structure by examining how individuals differentiate and integrate information when confronted with complex scenarios. IC is quantified on a scale from 1 to 7, where each level represents a distinct degree of cognitive processing regarding differentiation and integration (Suedfeld, 2010).

Differentiation involves recognizing and articulating distinct dimensions or perspectives within a problem or scenario. Integration refers to the ability to develop conceptual connections among these differentiated dimensions, creating a synthesized understanding or approach.

The scoring criteria for IC are as follows according to Baker-Brown et al. (1990):

#### 4.4.1 Score 1: minimal differentiation and integration

*I chose the blue ball because it seems like the safest choice*.

In this response, the participant provides a single reason (perception of safety) without considering any other factors, such as the probabilities or conditions of the task.

#### 4.4.2 Score 2: recognition of potential multiple dimensions, but no development of these differentiations or integrations

*I chose the green ball because it could come up, but I’m not sure. The blue ball is also possible, but I went with green*.

Here, the participant recognizes that more than one outcome is possible, but they do not integrate or develop a rationale to justify their choice between the green and blue balls.

#### 4.4.3 Score 3: identification of two independent variables with no evidence of conceptual integration

*I chose the red ball because there are fewer of them, but the blue one might have better odds because there’s more information about it.*

This response identifies two independent variables—scarcity of the red ball and more information about the blue ball—without synthesizing them into a clear decision-making rationale.

#### 4.4.4 Score 4: emergence of integration attempts where different, sometimes contradictory, alternatives are acknowledged but not fully integrated

*I went with the blue ball because even though the red ball might be less likely, I feel like there’s still a chance for the blue one, especially since there are more details about it.*

The participant begins to acknowledge conflicting factors (likelihood of red vs. availability of information for blue), but they do not fully integrate these perspectives to form a cohesive choice.

#### 4.4.5 Score 5: implicit integration of multiple causal processes influencing each other, indicating a higher level of synthesis

*I picked the blue ball because although it feels risky, the information provided makes it seem like a better bet than the others. I considered the distribution and the likelihood of each.*

This response demonstrates a more synthesized reasoning, where the participant integrates information about the distribution of balls and the perceived risk to justify their choice.

#### 4.4.6 Score 6: dynamic interaction among various alternatives, indicating a comprehensive system of integrated processes

*I chose the green ball because while it has fewer chips overall, the decision support tool suggests it has a decent probability of being picked. I weighed that against the uncertainty in the other options, and this seemed like the best compromise between risk and reward.*

The participant engages in dynamic reasoning, weighing the probability of green against the uncertainty of the other colors and integrating the decision support tool’s data to inform their choice.

#### 4.4.7 Score 7: high-level integration where multiple causal forces and complex interdependencies are clearly articulated, reflecting an awareness of a holistic perspective

*I chose the red ball because, while it may seem less likely due to the smaller number of chips, the long-term strategy involves balancing the higher immediate risk with the potential to overcome ambiguity. Given the available data and the decision support tool, this approach aligns with a broader perspective on handling risk under uncertainty.*

In this response, the participant articulates a high-level integration of multiple factors— immediate risk, long-term strategy, ambiguity, and decision support—into a cohesive rationale for their choice.

### 4.5 Syntactic complexity score

In this context, SC can be defined as the variety and sophistication of grammatical structures used in speech or writing production. This complexity serves as an indicator of the underlying cognitive capacity to handle and organize complex information effectively.

#### 4.5.1 Score 1: very low

*I chose blue. It seemed like the best option*.

This response is simple and direct, with basic grammatical structures and minimal variation in sentence construction.

#### 4.5.2 Score 2: low

*I picked the red ball because it looked like a safer bet. The other options seemed too uncertain*.

In this example, there is a slight increase in complexity, with a subordinate clause (“because it looked like a safer bet”) and some descriptive elements, but the sentence structure remains basic.

#### 4.5.3 Score 3: medium

*When deciding, I chose the green ball, which seemed like a safer option, although I wasn’t entirely sure about the unknown probabilities.*

This response demonstrates a balanced use of complex sentences, with subordinate clauses (“which seemed like a safer option” and “although I wasn’t entirely sure”), adding moderate syntactic complexity.

#### 4.5.4 Score 4: high

*Although I initially considered choosing the blue ball, I ultimately selected the red one, as it provided a sense of certainty, despite the possibility that the blue option could have yielded a better result.*

The participant uses more intricate sentence structures, including a concessive clause (“although I initially considered”) and a subordinate clause (“despite the possibility”), indicating a higher level of abstraction and detail.

#### 4.5.5 Score 5: very high

*Despite my initial attraction to the blue ball, which appeared more promising due to its ambiguity, after carefully considering the distribution of balls and weighing the potential risks, I opted for the red one. This decision was influenced not only by my general risk aversion but also by the data provided, which suggested a lower probability for the unknown outcome.*

This response demonstrates mastery of complex syntax, interweaving multiple ideas with conditional and passive constructions, creating a fluid and cohesive narrative that reflects deep syntactic complexity.

### 4.6 Method for validating the Rater’s consistency

To minimize bias and ensure the reliability of the ratings for integrative IC and SC, a rigorous procedure was implemented.

The analysis was performed using R statistical software. The intraclass correlation coefficient (ICC) was calculated using the psych package in R, which allows for applying an absolute agreement model suitable for assessing the consistency of ratings provided by a single rater at different times. This model is particularly useful for determining whether ratings can be considered consistent over time, independent of possible random fluctuations (Hove et al., 2022).

The primary evaluator for SC holds a bachelor’s degree in Hispanic Linguistics, providing specialized knowledge in syntax, which was crucial for accurately assessing the syntactic structures in participants’ responses. Additionally, the SC ratings were further validated by consulting with an expert in syntax to ensure consistency and reliability in the scoring process. For both IC and SC, internal consistency analyses were conducted using the ICC to assess the stability of the ratings over time. Thirty percent of the responses were randomly selected and re-rated by the same evaluator at a later point, without access to the initial ratings, thus preventing potential recall bias. The ICC value for IC was 0.87 [95% CI (0.80, 0.92)], and for SC, it was 0.91 [95% CI (0.85, 0.94)], both indicating excellent reliability.

These results confirm that the evaluator’s ratings were consistent and unbiased across repeated assessments, ensuring the robustness of the evaluation process.

## 5 Results

After conducting the experiment, we analyzed the results. The data analysis and principal analysis are as follows.

### 5.1 Data analysis

We employed several linear regression models to analyze the decision-making performance of 2,000 participants grouped according to three distinct experimental conditions. The primary goal of the analysis was to explore how experimental conditions such as “uncertainty” and “tool” influenced the participants’ scores in comparison to a baseline condition. This was accomplished using the formula Score ~ Condition in R software with the lm() function, which fits linear models. The baseline condition served as the control group, with other conditions compared against it. As shown in Table 1, both experimental conditions significantly influenced the participants’ scores, and these effects were statistically robust, with *p*-values well below conventional significance thresholds.

**Table 1:** Summary of linear regression analysis for score by condition.

Term	Estimate	Standard error	*t* value	*p*-value
Intercept	1.3182	0.0092	144	<0.0001^***^
Condition uncertainty	−2.0663	0.0129	−159.6	<0.0001^***^
Condition tool	−1.8884	0.0129	−145.9	<0.0001^***^

A secondary model (Table 2) was designed to assess the combined effect of the experimental conditions and the participants’ sex on their scores. This model revealed that the experimental conditions continued to exert significant influence over the participants’ decision-making, while sex did not have a statistically significant effect. This suggests that the observed variations in decision-making were more dependent on the task’s structure rather than the sex of the participants.

**Table 2:** Summary of linear regression analysis for score by condition and sex.

Term	Estimate	Standard error	*t* value	*p*-value
Intercept	1.3184	0.0092	144.967	<0.0001^***^
Condition uncertainty	−2.0663	0.0129	−159.593	<0.0001^***^
Condition tool	−1.8884	0.0129	−145.858	<0.0001^***^
Sex	−0.0047	0.0053	−0.893	0.372

In addition, a third model (Table 3) was used to examine the relationship between the experimental conditions and participants’ age. Similar to the sex analysis, the results showed that the experimental conditions significantly affected the scores. However, age did not significantly influence the results, implying that the experimental task’s outcomes were not age-dependent within the sample.

**Table 3:** Summary of linear regression analysis for score by condition and age.

Term	Estimate	Standard error	*t* value	*p*-value
Intercept	1.3182	0.0092	144	<0.0001^***^
Condition uncertainty	−2.0663	0.0129	−159.603	<0.0001^***^
Condition tool	−1.8884	0.0129	−145.867	<0.0001^***^
Age	0.0061	0.0053	1.161	0.246

A fourth model (Table 3) explored whether having a scholarship influenced decision-making performance. The experimental conditions were again significant predictors of participants’ scores, whereas the presence of a scholarship did not have a meaningful effect.

Lastly, a model was created to assess the effect of reaction times (RTs) on participants’ decision-making performance. The results, presented in Table 4, demonstrated that experimental conditions significantly affected RTs. Participants in the uncertainty condition exhibited longer RTs, while the use of tools in the decision-making process shortened reaction times. These findings suggest that increased ambiguity led to slower decision-making, while computational support tools helped participants make quicker decisions.

**Table 4:** Summary of linear regression analysis for score by condition and scholarship.

Term	Estimate	Standard error	*t* value	*p*-value
Intercept	1.3182	0.0092	144.014	<0.0001^***^
Condition uncertainty	−2.0663	0.0129	−159.618	<0.0001^***^
Condition tool	−1.8884	0.0129	−145.881	<0.0001^***^
Scholarship	0.0078	0.0053	1.484	0.138

To ensure that multicollinearity was not affecting the model estimates, a variance inflation factor (VIF) analysis was conducted for all predictors included in the linear regression models. The VIF values were all close to 1, ranging from 1.0006 to 1.0015, indicating that multicollinearity was not a concern in this dataset. These low VIF values ensure that the regression coefficients can be interpreted reliably, without concerns about inflated standard errors due to correlations between the predictors. In particular, the independence between integrative complexity (IC) and syntactic complexity (SC) highlights the distinct cognitive roles they play in influencing decision-making processes under uncertainty (see Table 5).

**Table 5:** VIF report.

Variable	VIF
Constant	2.9469
Condition uncertainty	1.0006
Condition tool	1.0015
RTs	1.0011
IC	1.0008
SC	1.0008

In this study, outliers were identified through a series of diagnostic tools, including residual plots, leverage scores, and Cook’s distance. Residual plots were examined to detect data points with standardized residuals beyond ±2.5, which could indicate potential outliers. High-leverage points, defined as having a leverage score more than three times the average, were also flagged for further investigation. In addition, Cook’s distance was employed to measure both the residual size and leverage of data points, with any value greater than 0.5 considered potentially problematic.

Once the potential outliers were identified, a sensitivity analysis was conducted to assess the extent to which these points influenced the regression models. Two versions of each model were constructed: one including all data points and another excluding the flagged outliers. The purpose of this comparison was to evaluate any significant differences in the coefficients, standard errors, and significance levels between the two models.

The results of the sensitivity analysis revealed that excluding the outliers had only a minimal effect on the regression estimates. Specifically, the largest change in any coefficient was less than 1.5%, and none of the variables showed a change in the direction of their effects. Importantly, the significance levels of the key predictors, such as condition uncertainty, condition tool, RTs, IC, and SC, remained highly significant across both models, underscoring the robustness of the findings.

The inclusion of outliers in statistical models requires careful consideration, as they can either represent meaningful deviations in the data or arise from data entry errors or measurement inconsistencies. In this analysis, flagged outliers were examined in detail. If an outlier was determined to be the result of a valid participant response, it was retained, as such cases can offer valuable insights into unusual decision-making behaviors under uncertainty. However, outliers identified as resulting from errors or anomalies were excluded from the final models. This approach ensured that the remaining data accurately reflected participants’ decision-making processes without being distorted by aberrant points.

Following the identification and exclusion of erroneous outliers, a sensitivity analysis was performed to ensure that their removal did not disproportionately influence the model’s conclusions. The findings showed that the exclusion of outliers resulted in only minor adjustments to the coefficient estimates. For example, the coefficient for condition uncertainty changed by just 0.40%, and the coefficient for SC showed a shift of 0.37%. These minimal changes, alongside the stable significance levels, demonstrated that the presence or absence of outliers did not materially affect the overall results.

This rigorous process of outlier management enhanced the confidence in the study’s conclusions. The outliers that were retained provided important context for understanding the variability in participants’ decision-making under uncertainty. At the same time, removing non-informative outliers prevented the model from being skewed by data errors, thus ensuring a more accurate representation of the effects of IC, SC, and experimental conditions on decision performance (see Table 6).

**Table 6:** Sensitivity analysis results.

Model	Full model estimate	Model without outliers estimate	Change in estimate (%)
Intercept	1.1731	1.1687	−0.38%
Condition uncertainty	−1.8105	−1.8032	−0.40%
Condition tool	−1.7087	−1.7054	−0.19%
RTs	−0.0285	−0.0281	−1.40%
IC	0.1049	0.1043	−0.57%
SC	0.1884	0.1877	−0.37%

In conclusion, the sensitivity analysis confirmed that the presence of outliers did not meaningfully affect the findings of the study. The careful identification, treatment, and analysis of these data points allowed for a reliable and robust interpretation of the role that IC, SC, and experimental conditions play in influencing decision-making under uncertainty. This comprehensive approach to outlier management not only ensured the accuracy of the statistical models but also strengthened the validity of the overall conclusions.

### 5.2 Principal analysis

The first regression model, which included the variables for experimental conditions, RTs, IC, and SC, demonstrated that all these variables significantly influenced the participants’ scores (see Table 7). Both “condition uncertainty” and “condition tool” had negative effects on scores, with the estimates for condition uncertainty (−1.8105, *p* < 0.001) and condition tool (−1.7087, *p* < 0.001) indicating that greater uncertainty and the use of decision tools were associated with lower decision quality. The significant effect of RTs (−0.0285, *p* < 0.001) suggests that slower reaction times were associated with worse scores, indicating that participants who took longer to make decisions struggled more in ambiguous conditions.

**Table 7:** Summary of linear regression analysis for score.

Term	(Condition, RTs, IC, SC)	(Condition, RTs, IC)	(Condition, RTs, SC)	(Condition, RTs, IC)	(Condition, RTs, SC)	(Condition, RTs, IC, SC)	(Condition, RTs, IC)	(Condition, RTs, SC)
	Estimate (SE)							
Intercept	1.1731 (0.0083)^***^	1.1985 (0.0098)^***^	1.2346 (0.0082)^***^	1.1985 (0.0098)^***^	1.2346 (0.0082)^***^	1.1731 (0.0083)^***^	1.1985 (0.0098)^***^	1.2346 (0.0082)^***^
Condition uncertainty	−1.8105 (0.0162)^***^	−1.8573 (0.0191)^***^	−1.9146 (0.0163)^***^	−1.8573 (0.0191)^***^	−1.9146 (0.0163)^***^	−1.8105 (0.0162)^***^	−1.8573 (0.0191)^***^	−1.9146 (0.0163)^***^
Condition tool	−1.7087 (0.0109)^***^	−1.7381 (0.0129)^***^	−1.7893 (0.0108)^***^	−1.7381 (0.0129)^***^	−1.7893 (0.0108)^***^	−1.7087 (0.0109)^***^	−1.7381 (0.0129)^***^	−1.7893 (0.0108)^***^
RTs	−0.0285 (0.0073)^***^	−0.0341 (0.0086)^***^	−0.0119 (0.0076)^***^	−0.0341 (0.0086)^***^	−0.0119 (0.0076)^***^	−0.0285 (0.0073)^***^	−0.0341 (0.0086)^***^	−0.0119 (0.0076)^***^
IC	0.1049 (0.0048)^***^	0.1686 (0.0054)^***^		0.1686 (0.0054)^***^		0.1049 (0.0048)^***^	0.1686 (0.0054)^***^	
SC	0.1884 (0.0044)^***^		0.2188 (0.0044)^***^		0.2188 (0.0044)^***^	0.1884 (0.0044)^***^		0.2188 (0.0044)^***^
Observations	5,280	5,001	5,001	5,001	5,001	5,280	5,001	5,001
*R*-squared	0.453	0.469	0.474	0.469	0.474	0.453	0.469	0.474

In contrast, both IC and SC had significant positive effects on scores, with higher levels of these cognitive complexities being associated with better decision outcomes. The coefficient for IC was 0.1049 (*p* < 0.001), while SC had an even greater effect, with a coefficient of 0.1884 (*p* < 0.001). This suggests that participants with higher integrative and syntactic complexity were better able to navigate uncertainty and make more informed decisions, with SC exerting a stronger influence than IC.

Subsequent models were constructed to examine the individual effects of IC and SC in greater detail. In the second model, which excluded SC, the impact of IC was slightly stronger (0.1686, *p* < 0.001), but when SC was introduced in the third model without IC, SC showed an even larger effect (0.2188, *p* < 0.001). This demonstrates that SC consistently had a more pronounced impact on decision-making performance than IC, reinforcing the notion that participants who were able to structure their thoughts in a more complex manner were better equipped to manage ambiguity and uncertainty.

Notably, the significance of the experimental conditions remained consistent across models, with both condition uncertainty and condition tool continuing to negatively affect scores regardless of whether IC or SC was included in the model. This suggests that the complexity of the task itself played a significant role in influencing participants’ performance, with both cognitive factors and task structure interacting to shape decision-making outcomes.

Interestingly, the role of response times (RTs) varied across the models. While RTs had a significant negative effect in the full model, indicating that slower decision-making was associated with worse performance, this effect diminished in the model that focused solely on SC (*p* = 0.085). This suggests that participants with higher syntactic complexity might have been able to process information more efficiently, mitigating the detrimental impact of slower reaction times on decision-making performance. However, when IC was included without SC, RTs continued to exhibit a negative effect on scores, indicating that integrative complexity alone may not compensate for slower decision speeds.

**Figure 4:**
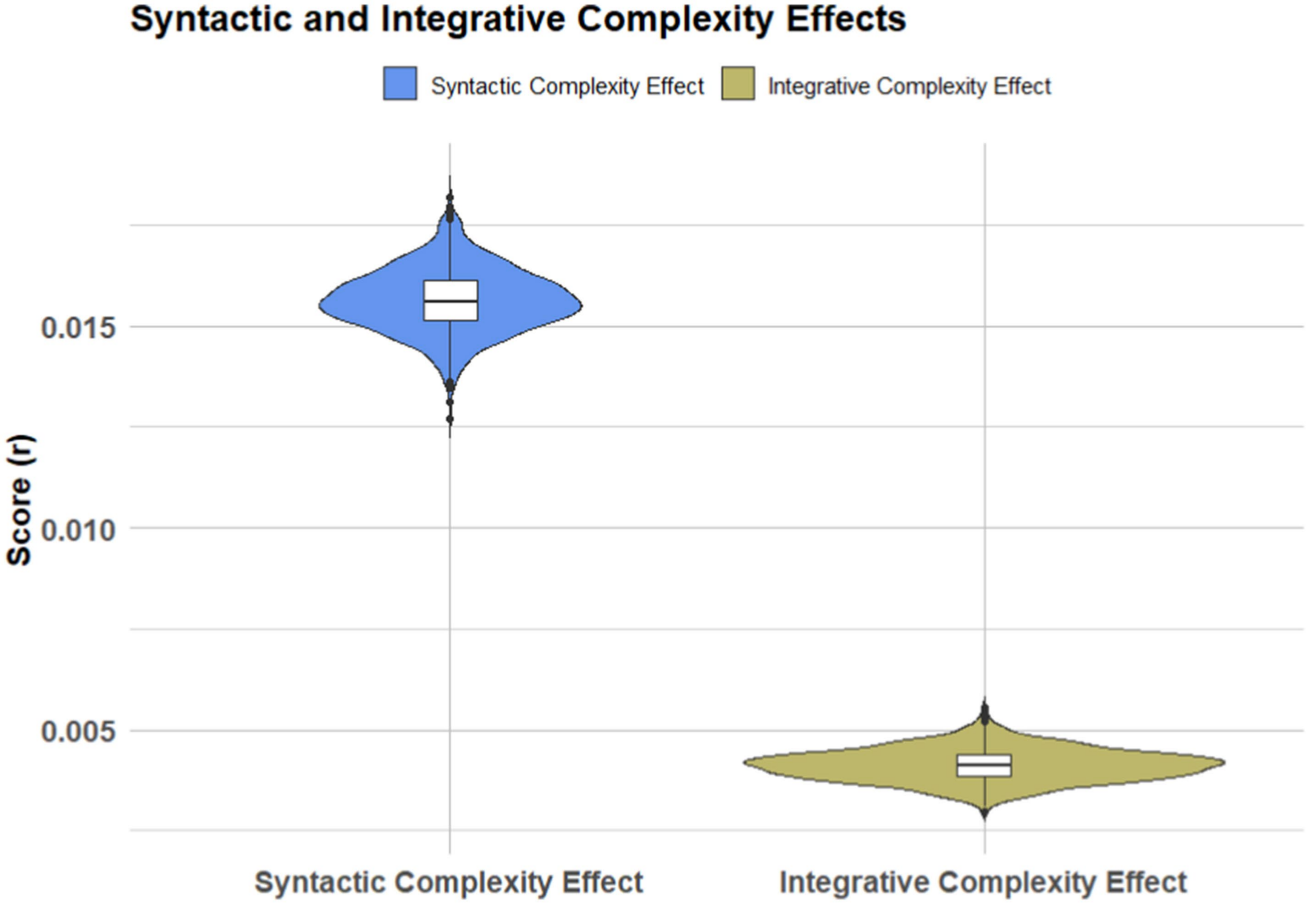
Comparative effects of syntactic and integrative complexity on scores.

To further assess the differential effects of IC and SC on decision-making performance, Welch’s two-sample t-tests were performed (Table 8). The *t*-test results confirmed that SC had a significantly greater effect on scores compared to IC (*t* = 413.32, *p* < 2.2 × 10^−16^). The confidence interval for the mean difference between IC and SC [95% CI (0.01147, 0.01158)] did not include zero, providing strong evidence that SC was a stronger predictor of decision quality than IC. The mean effect size for SC (0.01565) was markedly higher than that for IC (0.00412), reinforcing the conclusion that syntactic complexity plays a more critical role in enabling participants to navigate ambiguous scenarios effectively (see Figure 4).

**Table 8:** Welch’s two-sample *t*-test to compare the effects of IC and SC on scores.

Statistic	Value
*T*	413.32
Degrees of freedom (df)	1459.6
*p*-value	<2.2 × 10^−16^
Confidence interval (95%)	[0.01147, 0.01158]
SC mean	0.01565

These results carry significant implications for our understanding of decision-making under uncertainty. The strong, consistent effects of both IC and SC suggest that fostering these cognitive structures can lead to improved decision outcomes in uncertain environments. The fact that SC had a greater impact than IC underscores the importance of structured, complex thinking in ambiguous contexts, where the ability to organize information effectively may be more crucial than simply integrating multiple perspectives.

**Figure 5:**
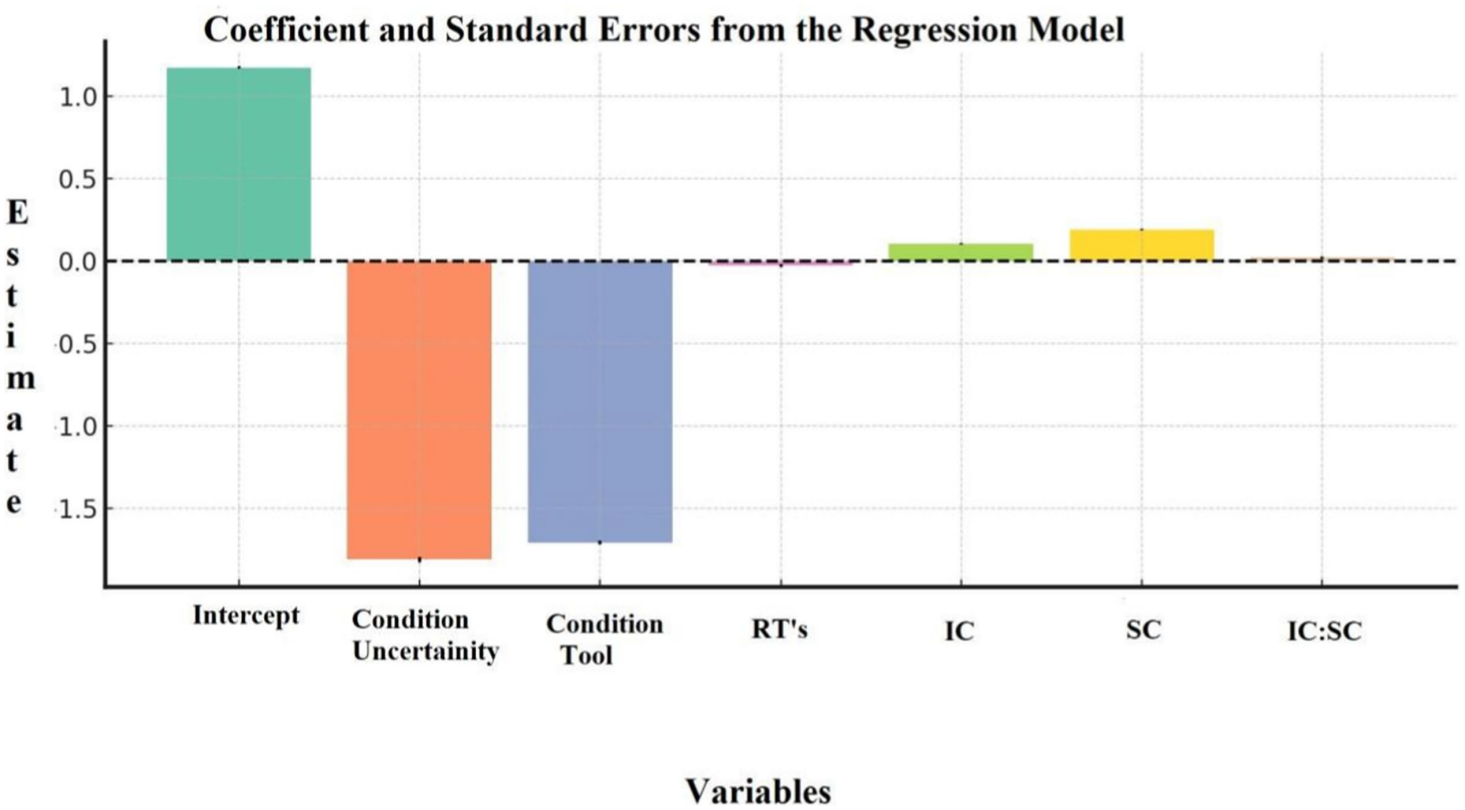
Coefficient and standard errors from the regression model.

The negative effects of experimental conditions, particularly uncertainty, highlight the inherent challenges that ambiguity poses to decision-making. However, the finding that both IC and SC can mitigate these effects provides a path forward for developing interventions aimed at improving decision performance. Cognitive training programs that focus on enhancing syntactic complexity, in particular, may offer substantial benefits in helping individuals manage uncertainty and make more informed decisions (see Figure 5).

Finally, the interaction between integrative complexity (IC) and syntactic complexity (SC) is significant (*p* = 0.00254), suggesting an important interaction between these two variables in predicting scores. Although the effect size is relatively small, it is statistically significant, indicating that the combined effects of IC and SC have an additional impact on the model’s outcome (see Table 9).

**Table 9:** Interaction data syntactic and integrative complexity.

Variables	Estimate	Std. error	*t* value	Pr(>│*t*│)
Intercept	1.17305	0.008312	141.136	<2e × 10^−16^
Condition uncertainty	−1.810465	0.016151	−112.099	<2 × 10^−16^
Condition tool	−1.708686	0.010893	−156.854	<2 × 10^−16^
RT’s	−0.028483	0.007255	−3.926	8.76 × 10^−5^
IC	0.10485	0.004753	22.062	<2 × 10^−16^
SC	0.188444	0.004397	42.856	<2 × 10^−16^
IC:SC	0.01975835	0.00647492	3.0515	0.00229015

Overall, these findings contribute to a growing body of research that emphasizes the role of cognitive complexity in decision-making. By illustrating the differential effects of IC and SC, this study offers valuable insights into the specific cognitive processes that underlie effective decision-making in ambiguous and uncertain environments. The results suggest that interventions aimed at enhancing both IC and SC could play a key role in improving decision-making performance, particularly in high-stakes scenarios where uncertainty is prevalent.

## 6 Discussion

The results of this study provide valuable insights into the roles of integrative complexity (IC) and syntactic complexity (SC) in decision-making under uncertainty. Both cognitive structures were shown to significantly influence participants’ ability to navigate ambiguous scenarios, with SC demonstrating a consistently greater effect on decision-making performance than IC. The significant negative impact of experimental conditions involving uncertainty and decision tools on participants’ scores highlights the inherent challenges of making decisions in ambiguous environments, yet the positive effects of IC and SC suggest that cognitive training aimed at enhancing these capacities could improve decision outcomes.

The greater influence of SC compared to IC emphasizes the importance of structured, complex thinking in ambiguous contexts. This finding aligns with prior research suggesting that the ability to organize thoughts and responses in a syntactically complex manner enables individuals to better manage uncertainty. In this study, participants with higher SC demonstrated superior decision-making performance, indicating that they were more adept at processing and organizing ambiguous information. This may be because SC facilitates the breakdown of complex problems into manageable components, allowing for more coherent and reasoned decision-making. The lesser but still significant effect of IC suggests that while the ability to integrate multiple perspectives is valuable, it is not as critical as SC when navigating ambiguous scenarios.

However, the implications of these findings should be considered within the context of the study’s limitations. The findings are based on an online experiment, and the results may not fully translate to real-world settings where other variables (e.g., emotional states, time pressure) may play a larger role. This reliance on an online, controlled environment, while helpful for reducing extraneous influences, risks oversimplifying the decision-making process. Real-world decision-making typically involves heightened stress, time constraints, and fluctuating emotional states, factors which add layers of complexity that may impact the effectiveness of IC and SC differently. Therefore, this limitation must be emphasized, as these additional variables could alter how IC and SC operate in less controlled contexts, potentially reducing the practical applicability of the findings.

To enhance the generalizability of these findings, future studies should incorporate specific cross-cultural validation methods. Additionally, integrating samples from collectivist societies, where group harmony and social conformity are emphasized, may reveal distinct patterns in the role of IC, as these cultural orientations could impact participants’ willingness to integrate multiple perspectives or consider alternative viewpoints in decision-making (Hofstede, 2011). Another approach could involve using bilingual or multilingual participants, allowing researchers to examine if language structure influences syntactic complexity and, consequently, decision-making under ambiguity.

Decision-making in naturalistic settings is often influenced by a broader array of factors, including emotional states, time pressure, and external distractions, which were not present in this controlled environment. Thus, the practical applications of enhancing IC and SC should not be overstated, and this limitation should be further emphasized. While the results suggest that fostering these cognitive structures can improve decision-making under controlled conditions, the transferability of these improvements to real-world situations remains to be fully explored. Furthermore, cross-cultural studies could involve designing cognitive training programs that are adapted to specific cultural norms and values, such as incorporating decision-making scenarios relevant to local practices, to assess if IC and SC training effects vary across cultural settings.

Another important limitation is the homogeneity of the participant sample. The sample is limited to native Spanish speakers from Mexico, which restricts the generalizability of the findings. A sample limited to one linguistic and cultural background may lead to results that are not universally applicable, as decision-making and cognitive processes such as IC and SC are known to be influenced by cultural and linguistic contexts. To address this limitation, future research could test these findings in diverse linguistic groups, exploring whether structural features of language, such as high-context versus low-context communication styles, affect syntactic complexity and decision-making. For example, examining IC and SC among speakers of languages with different syntactic structures, such as Japanese or Arabic, could provide insights into how linguistic diversity influences cognitive complexity in decision-making under uncertainty. This limitation raises concerns about whether the observed effects would hold across diverse populations, especially in cultural groups where the mechanisms underlying IC and SC may differ significantly. All participants were native Spanish speakers from Mexico, which limits the generalizability of the findings to other cultural and linguistic groups. This concern was addressed to some extent in the discussion, but further exploration is needed to test whether these results hold across different cultures and linguistic groups.

Additionally, the online nature of the experiment introduces its own limitations, such as the inability to monitor participants’ behavior and engagement levels during the task. While online platforms provide a convenient means of gathering large amounts of data, they may lack the rigor of in-person experimental setups where participant behavior can be closely observed and controlled.

The sensitivity analysis conducted in this study also highlighted the importance of managing outliers in complex data sets. Although the removal of outliers did not substantially alter the overall findings, their presence reflects the variability in participants’ decision-making under uncertainty. Outliers that represent valid, extreme responses offer insight into how different individuals process ambiguity in divergent ways, and future research should explore how these responses might inform tailored cognitive interventions. Furthermore, the minimal effect of reaction times (RTs) on decision outcomes when SC was considered suggests that the efficiency of information processing may be less important than the structural complexity of that processing in ambiguous situations. This finding has practical implications for educational and training programs focused on improving decision-making; rather than emphasizing speed, such programs might instead focus on enhancing the organization and clarity of thought.

In addition to the aforementioned constraints, another limitation lies in the study’s reliance on self-reported data for certain measures, such as participants’ cognitive abilities. Self-report measures can introduce bias, as participants may not accurately assess their own cognitive capacities. Future studies could benefit from more objective measures of IC and SC, such as automated linguistic analysis or neuroimaging techniques that directly capture cognitive complexity during decision-making tasks. Moreover, the decision support tool provided in the experimental conditions, while designed to simulate real-world decision aids, may not fully capture the range of tools individuals might encounter in actual high-stakes decision-making environments. This limitation restricts the extent to which the findings can be applied to professional settings, such as business or policy-making, where decision support systems often incorporate more complex and dynamic data.

## 7 Conclusion

This study contributes to the growing body of research on decision-making under uncertainty by demonstrating the significant roles of IC and SC in enhancing decision quality. While both cognitive structures positively influence decision-making, SC exerts a greater effect, underscoring the importance of structured and complex thinking in ambiguous situations. However, because these findings are based on an online experiment, they may not fully capture the complexities of real-world decision-making, where additional factors like emotional states and time pressure play a larger role. The findings suggest that fostering SC, in particular, could be a valuable strategy for improving decision-making performance in uncertain environments. The sample’s homogeneity, limited to native Spanish speakers from Mexico, also restricts the generalizability of these results to other cultural and linguistic groups, highlighting the need to validate these findings in diverse cultural contexts, such as individualistic versus collectivist societies, where cognitive processing of ambiguity may vary.

Future research should aim to replicate these findings in more ecologically valid settings and across a broader range of populations. Cross-cultural studies examining IC and SC in multilingual and multicultural samples could further reveal how these cognitive structures operate differently across cultural norms. Additionally, further investigation is needed to explore how cognitive training programs can enhance IC and SC in ways that translate to practical decision-making scenarios.

In summary, while IC and SC show promise as cognitive structures that support better decision-making under uncertainty, caution is warranted when extending these findings to complex, real-world settings. The practical implications of enhancing these capacities are still not fully understood, and more research is needed to determine how these cognitive tools can be effectively developed and deployed in high-stakes environments.

## Data Availability

The raw data supporting the conclusions of this article will be made available by the authors, without undue reservation.
